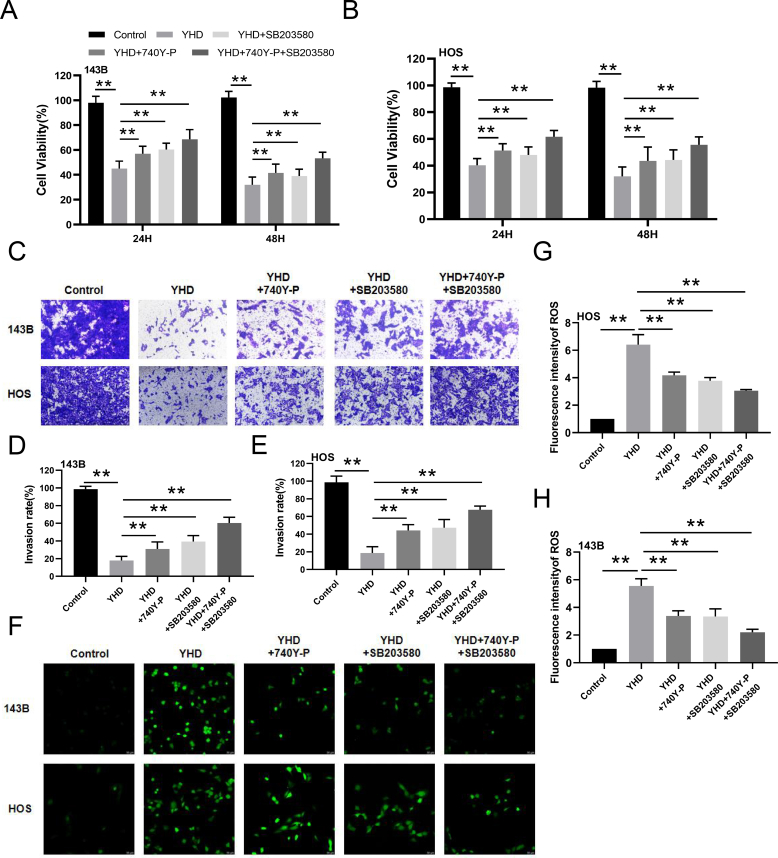# Network pharmacology reveals that *Yanghe Decoction* inhibits osteosarcoma progression via ROS-induced mitochondrial dysfunction and enhances cisplatin sensitivity

**DOI:** 10.1016/j.gendis.2025.101862

**Published:** 2025-09-20

**Authors:** Yanran Huang, Dagang Tang, Runhan Zhao, Jun Zhang, Xiao Qu, Ningdao Li, Yi Ren, Xiaoji Luo

**Affiliations:** a Department of Orthopaedic Surgery, Chongqing Municipal Health Commission Key Laboratory of Musculoskeletal Regeneration and Translational Medicine/Orthopaedic Research Laboratory, The First Affiliated Hospital of Chongqing Medical University, Chongqing 400016, China; b Chongqing General Hospital, Chongqing University, Chongqing, 401147, China; c Chongqing Hospital of Traditional Chinese Medicine, Chongqing 400011, China

**Keywords:** Mitochondrial function, Osteosarcoma, P38, PI3K/AKT, Yanghe decoction

## Abstract

Osteosarcoma (OS) is a highly aggressive bone malignancy with limited treatment options and frequent chemoresistance. *Yanghe Decoction* (YHD), a traditional Chinese medicine formula, has demonstrated anti-tumor potential, but its mechanisms in OS remain unclear. In this study, we employed a network pharmacology approach to identify 67 active components and 101 OS-related targets of YHD, with core targets including AKT1, TP53, MAPK14, and CASP3, mainly enriched in the PI3K/AKT and MAPK signaling pathways. Molecular docking confirmed strong binding affinities between representative compounds and these targets. Functional experiments revealed that YHD inhibited OS cell proliferation, migration, and invasion, and promoted apoptosis by elevating intracellular reactive oxygen species levels and inducing mitochondrial dysfunction. Mechanistically, YHD suppressed the PI3K/AKT pathway while activating p38 MAPK signaling. Importantly, YHD enhanced the sensitivity of OS cells to cisplatin, demonstrating a synergistic inhibitory effect *in vitro* and in an orthotopic OS mouse model. These findings suggest that YHD exerts its anti-osteosarcoma effects via reactive oxygen species-mediated mitochondrial disruption and pathway modulation, and may serve as a promising adjuvant to conventional chemotherapy.

## Introduction

Osteosarcoma (OS) is a common primary malignant tumor that often occurs in children and adolescents. In the majority of OS patients, it manifests in areas of rapidly growing bone, such as the metaphysis of long bones in the limbs.^1^ OS is characterized by its high malignancy and high metastatic rate. Most patients are diagnosed with metastases (typically to the lungs), which often results in high mortality and incidence rates.^2^^,^^3^ Common treatment methods for OS include amputation, limb-salvage surgery, chemotherapy, and immunotherapy.^4^ While these treatments have improved patient survival rates to some extent, they yield poor efficacy for metastatic patients. Chemotherapy often induces severe adverse reactions, thereby limiting its clinical application and therapeutic effectiveness.^5^^,^^6^ Hence, there is a necessity to explore new drugs with better treatment outcomes and minimal toxic side effects.

Traditional Chinese herbal medicine plays a significant role as an adjunct therapy in the prevention and treatment of OS. They can reduce the toxic side effects of chemotherapy, enhance sensitivity to conventional chemotherapy, and further modulate the patient's immune function, thereby exerting better anti-tumor effects.^7^^,^^8^ *Yanghe Decoction* (YHD) consists of seven medicinal ingredients: *Radix Rehmanniae praeparata*, *Cortex Cinnamomi*, *Ephedra sinica stapf*, *Semen brassicae*, *Zingiber offcinale Rose*, *Radix Rhizoma glycyrrhizae*, and *Colla cornus cervi*.^9^ Previous studies have shown that YHD has anti-inflammatory, analgesic, and anti-tumor effects.^9^, ^10^, ^11^ It can exert its anti-breast cancer effects through the phosphatidylinositol-3-kinase (PI3K)/protein kinase B (Akt) signaling pathway.^12^ It can inhibit autoimmune thyroiditis by improving NOD-like receptor 3 (NLRP3) inflammasomes and immune dysregulation^13^; and it can effectively alleviate symptoms of ankylosing spondylitis.^14^ It is evident that YHD has the potential to be developed as a novel source of traditional Chinese medicine for combating OS. However, its pharmacological activity research is not yet comprehensive enough, and its mechanism of action urgently needs clarification.

Mitochondria serve as the “powerhouse” of the cell and are also the primary source of cellular reactive oxygen species (ROS), playing crucial roles in biological processes, such as cell growth, proliferation, differentiation, and apoptosis.^15^ On one hand, mitochondria generate metabolic energy through oxidative phosphorylation and regulate pathways of apoptosis to maintain cellular homeostasis. On the other hand, ROS serve as important second messengers regulating cellular signaling.^16^ Elevated levels of intracellular ROS can lead to oxidative damage to DNA, proteins, and other molecules, thereby inducing cell cycle arrest, apoptosis, and senescence.^17^ Based on this mechanism, therapeutic strategies targeting mitochondrial dysfunction in cells are considered a promising approach for cancer treatment.

This study established a network pharmacology model of YHD and systematically analyzed the mechanism of YHD in the treatment of OS. Through *in vitro* and *in vivo* experiments, the anti-OS cell effects of YHD and its underlying mechanisms were explored. It was found that YHD could induce an increase in ROS in OS cells, thereby causing mitochondrial dysfunction. We also elucidated its relationship with the PI3K/AKT and P38 signaling pathways, providing a theoretical basis for subsequent clinical applications.

## Material and methods

### Material

Fetal bovine serum, Dulbecco's Modified Eagle Medium (DMEM), and Minimum Essential Medium (MEM) were purchased from GIBCO (USA). Penicillin-streptomycin, trypsin, crystal violet staining solution, CCK8, annexin V-FITC/PI cell apoptosis detection kit, BCA protein concentration assay kit, ECL luminescence reagent kit, 2′,7′-dichlorodihydrofluorescein diacetate (DCFH-DA), Hoechst 33,258, and JC-1 staining solution were purchased from Beyotime Biotechnology (China). Transwell chambers were purchased from Corning (USA). Matrigel matrix gel was purchased from BD (USA). Radioimmunoprecipitation assay lysis buffer was purchased from Invitrogen (USA). MitoSOX Red superoxide indicator was purchased from Thermo Fisher (USA). ATP assay kit was purchased from Solarbio (China). qPCR SYBR Green Master Mix and reverse transcription kit were purchased from Vazyme (China). Seahorse XFp Cell Mito Stress Test kit was purchased from Agilent (USA). Proliferating cell nuclear antigen (PCNA) (#F0018), B-cell lymphoma 2 (Bcl-2)-associated X protein (Bax) (#F0037), and glyceraldehyde 3-phosphate dehydrogenase (GAPDH) (#F0003) antibodies were purchased from Selleck (USA). Cyclin B (#4138), Vimentin (#5741), N-cadherin (#13116), p38 (#9212), and p-p38 (#4511S) antibodies were purchased from Cell Signaling Technology (USA). Cytochrome c (CYT-C) (#AF0146), cleaved-PARP (#AF7023), Bcl-2 (#AF6139), cleaved-caspase 9 (#AF5240), cleaved-caspase 3 (#AF7022), matrix metalloproteinase 2 (MMP2) (#AF5330), MMP7 (#AF0218), peroxisome proliferator-activated receptor-γ-coactivator 1α (PGC-1α) (#AF5395), nuclear respiratory factor 1 (NRF1) (#AF5298), NRF2 (#AF0639), mitochondrial transcription factor A (TFAM) (#AF0531), PI3K (#AF6241), p-PI3K (#AF3242), AKT (#AF6261), p-AKT (#AF0016), extracellular signal-related kinase (ERK) (#AF0155), Jun N-terminal kinase (JNK) (#AF6318), p-ERK (#AF1015), p-JNK (#AF3318), and Snail (#AF6032) antibodies were purchased from Affinity Biosciences (USA).

### Preparation of YHD-containing serum

YHD is composed of 3 g of *Glycyrrhiza uralensis*, 30 g of *Rehmanniae Radix Praeparata*, 2 g of *Ephedra vulgaris*, 2 g of *Zingiber officinale Roscoe*, 3g of *Cortex Cinnamomi Cassiae*, 9 g of *Colla Cornus Cervi*, and 6 g of *Sinapis Semen*. These medicinals were obtained from the Chinese Medicine Pharmacy of Chongqing Traditional Chinese Medicine Hospital (Chongqing, China). The identities of all medicinals have been confirmed by Professor Renyi of Chongqing Traditional Chinese Medicine Hospital. Male Sprague–Dawley rats (weighing 200–220 g) were purchased from the Animal Research Center of Chongqing Medical University. After one week of adaptive feeding, they were divided into the control group and the YHD group. The rats were administered ultrapure water or YHD (10 g/kg) by gavage, twice daily for 10 consecutive days. One hour after the final dose, blood was collected from the abdominal aorta. The blood was centrifuged at 4 °C for 30 min to collect the upper serum layer, which was then inactivated in a 56 °C water bath for 30 min, sterilized by filtration through a 0.22 μm microporous membrane, and stored at −80 °C for later use (approval number: IACUC-CQMU-2024-04063).

### Ultra-performance liquid chromatography–tandem mass spectrometry analysis

Components of YHD-containing serum were analyzed using the Vanquish UHPLC system (Thermo Fisher Scientific, USA). The temperature of the chromatographic column was maintained at 40 °C. The flow rate was set at 0.3 mL/min, with an injection volume of 2 μL. In the positive mode of mass spectrometry, the mobile phase consisted of 0.1% formic acid acetonitrile solution (volume ratio) and 0.1% formic acid aqueous solution (volume ratio). In the negative mode of mass spectrometry, the analytes were carried using acetonitrile and 5 mM ammonium formate. Parameters for the Orbitrap Exploris 120 (Thermo Fisher Scientific, USA) included: sheath gas pressure of 40 arb, auxiliary gas flow rate of 10 arb, spray voltage of 3.50 kV for positive ion mode, and spray voltage of −2.50 kV for negative ion mode. The collected data were analyzed using the R XCMS package.

### Target screening of the active components of YHD and OS

Predict potential targets of compounds in YHD using the Traditional Chinese Medicine Systems Pharmacology Database and Analysis Platform (TCMSP) database, PharmMapper database, and SwissTargetPrediction database. The target points corresponding to the compounds were screened through the Drug Bank database and sorted out, and duplicate items were deleted. At the same time, the UniProt database was used to define the species as “homo sapiens”, and the gene information of the target was calibrated to obtain the standardized target name. Taking “osteosarcoma” as the keyword, the GeneCards database (http://www.genecards.org/), DisGeNET database (https://www.disgenet.org/), and OMIM database (http://omim.org) were used to search for OS-related targets. The targets were taken with the correlation score ≥5 in the GeneCards search results, and were merged with the search results of the DisGeNET database and OMIM database, and then the targets related to OS after removing the duplication were obtained. The online software Venny 2.1 (https://bioinfogp.cnb.csic.es/tools/venny/) was used to intersect the targets of the active ingredients of YHD and the related targets of OS, and the potential targets of YHD for the treatment of OS were obtained.

### GO and KEGG enrichment analysis

The DAVID database (https://david. ncifcrf. gov/) is used for Gene Ontology (GO) and Kyoto Encyclopedia of Genes and Genomes (KEGG) enrichment analysis for intersection targets. The biological functions of gene targets were analyzed by GO, including cellular components, molecular functions, and biological processes. The related pathways involved were obtained through KEGG pathway analysis, and the mechanism of YHD in the treatment of OS was elucidated from the perspective of molecular biology.

### Molecular docking

Molecular docking was applied to the main 20 active components of YHD and core targets. The 3D structure of core targets tumor protein p53 (TP53) (5O1F), AKT1 (1UNP), caspase 3 (CASP3) (7RNF), cyclin D1 (CCND1) (6P8G), epidermal growth factor receptor (EGFR) (1M17), phosphatidylinositol-4,5-bisphosphate 3-kinase catalytic subunit gamma (PIK3CG) (2A5U), myelocytomatosis viral oncogene homolog (MYC) (1NKP), mitogen-activated protein kinase 14 (MAPK14) (6SFO), and BCL2 (6GL8) were downloaded from the Protein Data Bank (PDB) database (https://www.rcsb.org/). Pymol 2.5.1 software was used to remove small molecules, AutoDock 4.2.6 was then used to remove water from the protein and replace it with hydrogen, and the structure was saved as a PDBQT protein receptor file. The three-dimensional structure diagram of the active ingredient was downloaded from the PubChem online database (https://pubchem.ncbi.nlm.nih.gov/), and the processing steps were the same as above with AutoDock 4.2.6. The active components are ligands, and the ten core genes are receptors. Then, the processed protein was docked with small molecules through autodocking, and the docking binding energy between the component and the target protein was calculated using the Auto Dock Vina 1.1.2 tool. Lower binding energy of molecular docking conformation and more stable binding conformation represent a greater possibility of reflecting the binding between receptor molecules and ligands.

### Cell culture

Human osteosarcoma cell lines (HOS, 143B), human bone marrow stromal cells (HS-5), human proximal renal tubular epithelial cells (HK-2), and human normal liver cells (LO2) were all purchased from the American Type Culture Collection (ATCC). HOS cells were maintained in MEM, while the other mentioned cells were cultured in DMEM. All media were supplemented with 10% fetal bovine serum, 100 U/mL penicillin, and 100 μg/mL streptomycin. All cells were cultured in a constant temperature incubator at 37 °C with 5% CO_2_ and saturated humidity. Cell groups included: 20% control rat serum (control), blank control group (blank), 10% medicated serum group (10% YHD), 15% medicated serum group (15% YHD), and 20% medicated serum group (20% YHD).

### Flow cytometry analysis

Cells were seeded at a density of 1 × 10^6^ cells per well in 6-well plates overnight and then cultured in a 37 °C, 5% CO_2_ incubator. After 24 h, the cells were digested with trypsin, centrifuged, and collected. After gently washing twice with phosphate-buffered saline solution, 500 μL of 1 × binding buffer, 5 μL of annexin V, and propidium iodide were added to each well. The cells were mixed thoroughly and stained in the dark at room temperature for 15 min. To determine the cell cycle distribution, cells were collected and fixed at 4 °C overnight with 500 μL of 75% ethanol, stained with propidium iodide solution, and then analyzed by flow cytometry.

### CCK8 assay

Cells in the logarithmic growth phase were seeded in 96-well cell plates at a concentration of 3000. After the cells adhered to the wall, YHD was added according to the above grouping and cultured for 24 h and 48 h. After the incubation, 10 μL of CCK8 was added to each well, and the incubation was continued for 4 h. The absorbance value of each well was measured at a wavelength of 450 nm on a microplate reader.

### Scratch healing assay

OS cells in the logarithmic growth phase were seeded in 6-well plates. After the cells were confluent, the surface of the OS cells was scratched with a 10 μL pipette tip, followed by washing with phosphate-buffered saline three times. The different concentrations of YHD were added to the OS cells. Scratch widths were recorded with a microscope at 0, 12, and 24 h. Scratch healing rate = (0 h scratch width – 24 h scratch width)/0 h scratch width × 100%.

### Transwell assay

OS cells in the logarithmic growth phase were collected and resuspended in serum-free DMEM. OS cells (2 × 10^4^) were added to the upper chamber of a Transwell plated with Matrigel. 500 μL of complete medium containing different concentrations of YHD was added to the lower chamber, followed by incubation at 37 °C and 5% CO_2_ for 48 h. The Transwell chamber was taken out, and the cells in the upper chamber that did not pass through the membrane were wiped off with a cotton swab, fixed in paraformaldehyde for 10 min, and stained with 0.1% crystal violet for 30 min. Observing and taking pictures under a microscope, five fields of view were randomly selected from each chamber.

### Colony formation assay

OS cells were seeded at a density of 1000 cells per well in 6-well plates. After the cells adhered to the well, they were treated according to the predetermined experimental groups and cultured in a 37 °C, 5% CO_2_ environment. The culture was continued for 10 days until colonies visible to the naked eye formed. After discarding the original culture medium, the cells were fixed for 20 min, washed twice with phosphate-buffered saline, stained with crystal violet for 30 min, rinsed, air-dried, and then photographed.

### ROS and mitochondrial superoxide detection

DCFH-DA was used to detect total ROS, and MitoSOX Red mitochondrial superoxide indicator was used to detect mitochondrial superoxide. OS cells were stained with 10 μM DCFH-DA and 5 μM MitoSOX Red mitochondrial superoxide indicator working solution. The cells were incubated at 37 °C for 10 min. After washing three times with phosphate-buffered saline, the cell nuclei were stained with Hoechst, and images were finally obtained through a fluorescence microscope.

### Mitochondrial membrane potential and oxygen consumption rate measurement

JC-1 staining was used to assess mitochondrial membrane potential changes. Briefly, OS cells were seeded on confocal dishes. After treatment, JC-1 working solution was added and incubated in the dark at 37 °C for 20 min. Cells were then observed and imaged using a laser confocal microscope. Under increased mitochondrial membrane potential, JC-1 aggregated in the mitochondrial matrix, forming JC-1 aggregates that emitted red fluorescence. Conversely, under low mitochondrial membrane potential, JC-1 did not aggregate in the mitochondrial matrix, resulting in the emission of green fluorescence as monomers.

Oxygen consumption rate was measured using the Seahorse Extracellular Flux (XFe24) analyzer (Seahorse Bioscience, USA). Briefly, OS cells were seeded in XFe24 plates and treated according to predefined groups. Before measuring oxygen consumption rate, the cell culture medium was replaced with XF base medium. After the addition of 2 μM oligomycin, 1.5 μM carbonyl cyanide-4 (trifluoromethoxy) phenylhydrazone (FCCP), 2 μM rotenone, and 2 μM antimycin A, oxygen consumption rate was measured using the analyzer.

### Mitochondrial damage detection

Cellular DNA was extracted using the phenol-chloroform method, and DNA concentration was measured. Real-time quantitative PCR was used for amplifying the test samples under the following conditions: initial denaturation at 95 °C for 10 min, denaturation at 95 °C for 15 s, annealing and extension at 60 °C for 34 s, for a total of 40 cycles. For mitochondrial DNA, the upstream primer was 5′-AATATTAAACACAAACTACCACCTACC-3′, and the downstream primer was 5′-CCGTAGCCTCATGAGCTGTT-3′. The 2^–ΔΔCT^ method was used to analyze mitochondrial DNA levels.

### ATP content measurement

The ATP content was measured by extracting samples according to the instructions of the ATP assay kit. Following the kit's protocol, the supernatant was added along with the diluent and working solution for chemiluminescence measurement. ATP content was detected using a spectrophotometer.

### Western blotting

OS cells in the logarithmic growth phase were seeded in 6-well cell plates. After the cells adhered to the wall, YHD was added according to the above grouping and cultured for 24 h. After the culture, the total protein of each cell sample was extracted with cell lysate, and the protein concentration was determined by the BCA method. After the protein samples were separated by 10% SDS-PAGE, the proteins were transferred to PVDF membranes, blocked with phosphate-buffered saline Tween-20 containing 5% bovine serum albumin for 1 h, and incubated at 4 °C overnight after the addition of specific primary antibodies. After washing the membrane with Tris-buffered saline Tween-20 three times, the corresponding secondary antibodies were added and subjected to incubation on a shaking table at room temperature for 1 h, followed by washing of the membrane with Tris-buffered saline Tween-20 three times. Finally, the protein bands were imaged and analyzed using the Gel Imaging Analyzer System.

### Transcriptome sequencing

Total RNA was extracted from 143B cells with or without YHD treatment for RNA sequencing. The RNA samples were sent to Shanghai Majorbio Bio-pharm Technology Co., Ltd. (Shanghai, China) for sequencing. RNA sequencing was performed using the NovaSeq X Plus platform (Illumina, California, USA). After obtaining the sequencing data, probe annotation was conducted; in cases of duplicate probes, the one with the highest average expression was retained. Following probe annotation, bioinformatics analyses were carried out. First, principal component analysis was performed to assess differences in gene expression patterns between the control and YHD groups. Next, differential expression analysis was conducted using the “DESeq2” R package. Subsequently, GO and KEGG enrichment analyses were performed to investigate the biological functions of differentially expressed genes. Finally, Gene Set Enrichment Analysis (GSEA) was applied to explore pathway differences between the two groups.

### Animal experiments

BALB/c nude mice (female, 4 weeks old) were purchased from Beijing Huafukang Biotechnology Co., Ltd. (China) and bred in a specific pathogen-free condition at the Department of Animal Center of Chongqing Medical University. The animal experiment was approved by the Animal Ethics Committee of the Chongqing Medical University (No. IACUC-CQMU-2024-04063). After anesthesia, 80 μL of 143B cells (2 × 10^6^) were injected into the tibial plateau of nude mice. Three days after injection, the mice were randomly divided into four groups (*n* = 6 per group): blank, YHD low dose (YHDL, 10 g/kg), YHD medium dose (YHDM, 20 g/kg), YHD high dose (YHDH, 30 g/kg). In the combined medication experiment with YHD and cisplatin (CDDP), mice were randomly divided into four groups (*n* = 6 per group): control group, YHD group (20 g/kg), CDDP group (8 mg/kg), and YHD/CDDP combination group. The mice were administered different doses of medication by gavage every other day. Meanwhile, the body weight and tumor size of nude mice were recorded. After 21 days, animals were killed with 2 % pentobarbital anesthetic and high concentrations of carbon dioxide. Relevant tissues from the mice were collected for analysis.

### Hematoxylin and eosin staining and immunohistochemistry analysis

Tissues were fixed in 4% paraformaldehyde, embedded in paraffin, and serially prepared into 5 μm sections. Sections were then stained with hematoxylin and eosin. For immunohistochemistry analysis of tumor tissue, the sections, after antigen retrieval, were incubated with the corresponding primary antibody. Subsequently, the sections were incubated with a horseradish peroxidase-conjugated secondary antibody. After staining with 3,3′-diaminobenzidine (DAB), the sections were imaged for analysis under a microscope.

### Statistical analysis

Statistical analysis was performed using GraphPad Prism 9.1.2. Data were expressed as mean ± standard deviation. The *t*-test was used for comparison between two groups, and the one-way ANOVA test was used for comparison between multiple groups. All data were from at least three independent experiments. *p*-values < 0.05 were considered statistically significant.

## Results

### Screening and target prediction of active compounds in YHD

Through ultra-performance liquid chromatography–tandem mass spectrometry analysis, we obtained the total ion chromatograms of YHD-containing serum, which displayed abundant chromatographic peaks in both positive and negative ion modes (Fig. S1A and S1B). We identified 67 active compounds (Table S1). Subsequently, we collected targets corresponding to these active components from databases and obtained 279 unique targets after removing duplicates (Table S2). We retrieved OS-related targets, and after removing duplicates, we obtained 1239 OS-related disease targets (Table S2). Using the online tool Venny 2.1.0, we identified the intersection of targets corresponding to active components of YHD and OS-related gene targets, eventually identifying 101 crossover targets as detailed in Table S2 (Fig. 1A). Next, we performed protein–protein interaction network analysis on these crossover targets and identified 10 core targets, including AKT1, MYC, TP53, BCL2, CASP3, CCND1, PTEN, hypoxia-inducible factor 1-alpha (HIF1A), estrogen receptor 1 (ESR1), and EGFR (Fig. S1C). These core targets may be key actionable targets for YHD treatment of OS. Further network clustering analysis using the MCODE plugin in Cytoscape 3.9.1 identified three main clusters, each encompassing the aforementioned 10 core targets (Fig. S1D–S1F), validating the accuracy of our central network screening results. Additionally, we conducted GO analysis and found significant enrichment in biological processes, such as ROS generation, cell apoptosis, cell proliferation, and mitochondrial function (Fig. 1B–D). Through KEGG pathway analysis, we discovered that the potential therapeutic action of YHD on OS was mainly regulated through the PI3K/AKT and MAPK signaling pathways (Fig. 1E and F). Further, combining chemical components, shared target genes, and the top 20 KEGG pathways, we constructed a “component-target-pathway-disease” network diagram using Cytoscape to visually display the potential therapeutic mechanisms of YHD on OS (Fig. 1G). This provides a systematic perspective for understanding the comprehensive impact of YHD on the treatment of OS. To further validate the interaction between the identified core targets and the active compounds of YHD, we performed molecular docking analysis. We selected 20 main active components and 10 key targets for molecular docking to elucidate the key pathways of YHD active components in treating OS. The heatmap shows that the main components of YHD have good binding activity with key targets in OS, and visualizes the docking results of key components with AKT1, BCL-2, CCND1, and MAPK14 (Fig. 1H and I).

**Figure 1:**
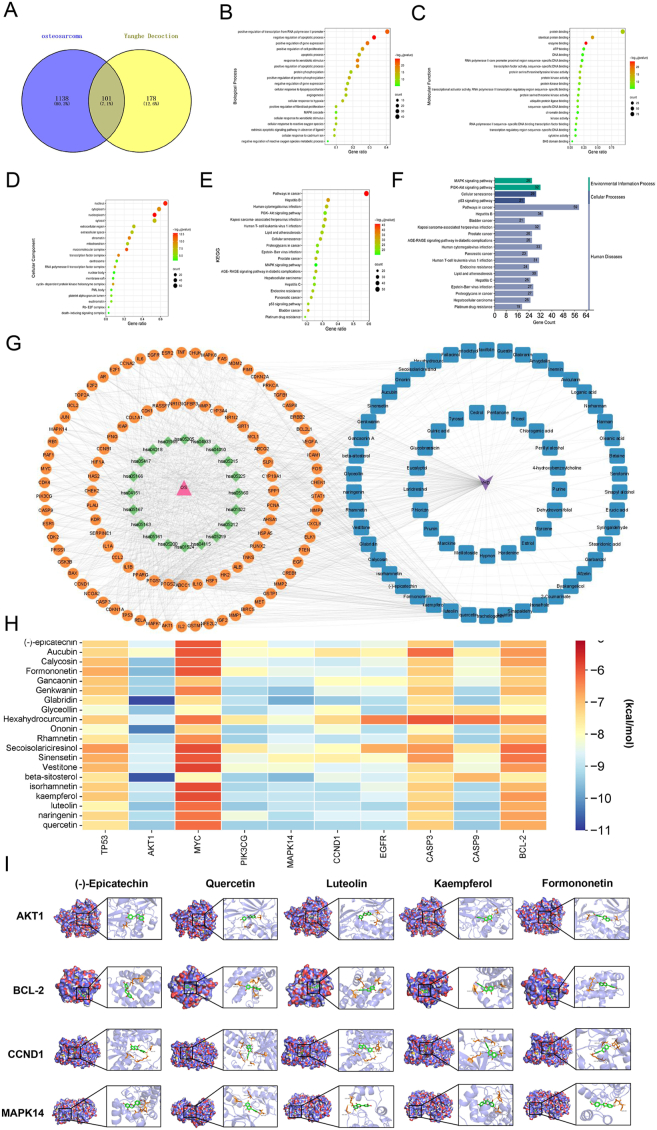
Identification of active compounds and target prediction in YHD. **(A)** Venn diagram of the target of YHD and the target of osteosarcoma. **(B**–**D)** Gene Ontology (GO) enrichment analysis results. **(E, F)** Kyoto Encyclopedia of Genes and Genomes (KEGG) enrichment analysis results. **(G)** The component-target-pathway-disease network implicated in the mechanism of YHD in osteosarcoma treatment. The triangles represent osteosarcoma, the diamonds represent pathways, the circles represent key genes, and the squares represent the active ingredients of YHD. **(H)** Heatmap of molecular docking score. A binding energy heatmap with a bluer color indicates a more stable binding. **(I)** Molecular docking visualization between the active components of YHD and key targets.

### YHD selectively inhibits OS cells without affecting the viability or apoptosis of normal human cells

To explore the effects of YHD on OS cells, we treated OS cells (143B and HOS cells) with 5%, 10%, 15%, 20%, and 25% YHD-containing serum for 24 h. Based on the results of the CCK8 assay, we selected 10%, 15%, and 20% YHD-containing serum for subsequent experiments (Fig. S2A). We then treated human normal liver cells (LO2), human renal cortical proximal tubular epithelial cells (HK2), and human bone marrow stromal cells (HS5) with YHD to explore its toxic side effects. CCK8 assay results showed that YHD had no inhibitory effect on the proliferation of LO2, HS5, and HK2 cells (Fig. S2B). Similarly, flow cytometry results indicated that YHD did not promote apoptosis in LO2, HS5, and HK2 cells (Fig. S2C and S2D). Overall, YHD can affect the activity of OS cells, while it has no impact on the proliferation and apoptosis of normal human cells.

Given its selective effect on OS cells without harming normal cells, we further investigated the antiproliferative mechanisms of YHD in OS cells. We first explored the impact of YHD on the proliferation of OS cells (143B and HOS). From colony formation experiments, we observed that YHD reduced the colony-forming ability of OS cells (Fig. 2A and B). Further, after treating OS cells with YHD for 24 and 48 h, CCK8 assay results indicated that YHD inhibited the proliferation of 143B and HOS cells in a dose-dependent manner (Fig. 2C and D). We then assessed the effects of YHD on the cell cycle of OS cells using flow cytometry and found a significant increase in OS cells in the G2/M phase after YHD treatment (Fig. 2E–G). Additionally, we detected proliferation-related protein PCNA and cell cycle-related protein cyclin B using Western blotting, with results consistent with the cell cycle changes observed in flow cytometry (Fig. 2H and I). Therefore, we speculate that YHD can effectively inhibit the proliferation of OS cells and may exert its anti-tumor effects by interfering with the G2/M phase of the cell cycle.

**Figure 2:**
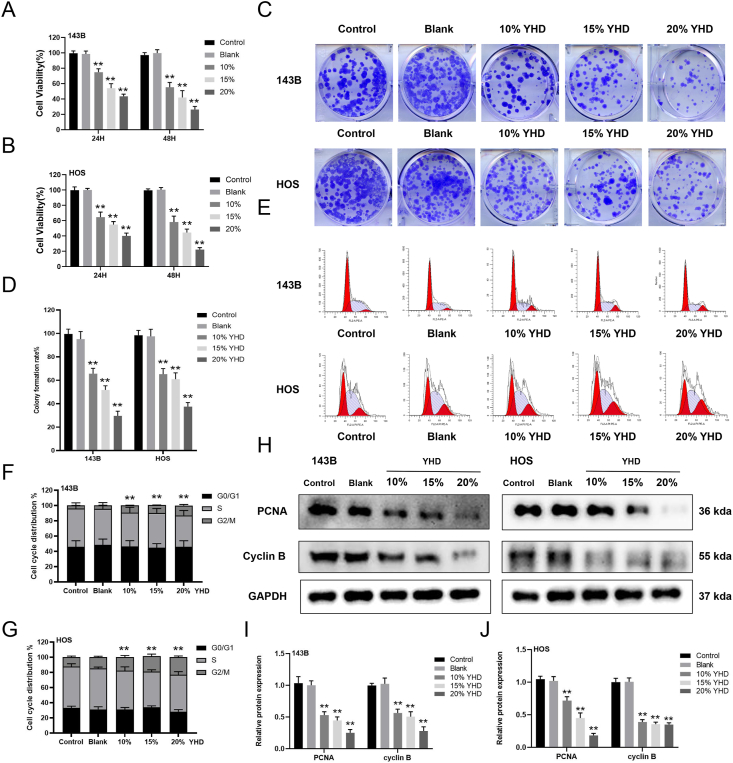
YHD selectively inhibits osteosarcoma (OS) cells without affecting the viability or apoptosis of normal human cells. **(A, B)** CCK8 assay detected the effect of YHD on the viability of OS cells at 24 h and 48 h. **(C, D)** Colony formation assay detected the effect of YHD on the colony-forming ability of OS cells. **(E**–**G)** Flow cytometry was used to detect the effect of YHD on the cell cycle of OS cells. **(H**–**J)** Western blot analysis detected the effect of YHD on the levels of proliferation-related proteins in OS cells. Data were presented as mean ± standard deviation (*n* = 3). ∗*p* < 0.05 and ∗∗*p* < 0.01 versus the blank group.

### YHD can inhibit the migration and invasion of OS cells

**Figure 3:**
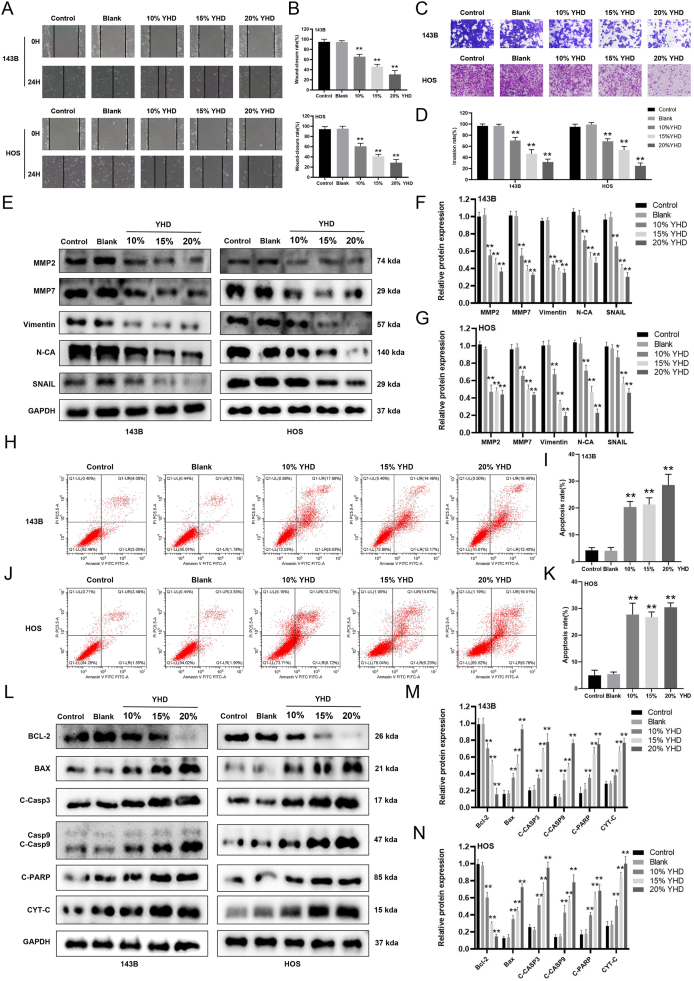
YHD can inhibit the migration and invasion of osteosarcoma (OS) cells, and promote their apoptosis. **(A, B)** Scratch healing assay showed that YHD inhibited the migration of OS cells. **(C, D)** Transwell assay showed that YHD inhibited the invasion of OS cells. **(E**–**G)** Western blot analysis detected the effect of YHD on the levels of proteins related to migration and invasion in OS cells. **(H–K)** Flow cytometry was used to detect the effect of YHD on apoptosis in OS cells. **(L**–**N)** Western blot analysis detected the effect of YHD on the levels of apoptosis-related proteins in OS cells. Data were presented as mean ± standard deviation (*n* = 3). ∗*p* < 0.05 and ∗∗*p* < 0.01 versus the blank group.

The next part of our study focused on investigating the effects of YHD on the migration and invasion capabilities of OS cells. Initially, the migration capacity of OS cells was evaluated using a wound healing assay. The results showed that YHD treatment significantly reduced the migration ability of OS cells (Fig. 3A and B), suggesting that YHD may inhibit cell migration. Additionally, we used Transwell assay to evaluate the cells' invasive capabilities. The results demonstrated that YHD treatment significantly reduced the number of OS cells that could penetrate the Matrigel coating and migrate to the upper chamber (Fig. 3C and D). These findings indicate that YHD has the potential to inhibit the migration and invasion of OS cells.

Epithelial–mesenchymal transition (EMT) refers to the transformation from epithelial to mesenchymal cells, which is one of the key steps in tumor cells acquiring invasive and metastatic capabilities. Through Western blot analysis, we found that YHD significantly down-regulated proteins that promote EMT, such as Snail, Vimentin, and N-cadherin, and up-regulated the anti-EMT protein E-cadherin (Fig. 3E–G). Overall, these experimental results indicate that YHD effectively reduces the migration and invasion capabilities of OS cells by inhibiting EMT.

### YHD promotes apoptosis in OS cells

We further investigated the effect of YHD on the apoptosis of OS cells. Initially, OS cells were cultured in serum containing different concentrations of YHD for 48 h. Apoptosis rates were measured using flow cytometry, and we observed that YHD significantly increased the apoptosis rate of OS cells (Fig. 3H–K). Subsequently, we measured the levels of apoptosis-related proteins via Western blotting. The results showed that YHD treatment significantly down-regulated key proteins in the mitochondrial apoptosis pathway, such as BAX and CYT-C, and promoted the activation of the caspase family proteins (cleaved-caspase 3, cleaved-caspase 8, and cleaved-caspase 9), and up-regulated the expression of the anti-apoptotic protein BCL-2 (Fig. 3L–N). These findings indicate that YHD may promote the activation of the mitochondrial pathway and thus lead OS cells towards programmed cell death by regulating the expression of these proteins.

### YHD inhibits OS cells by increasing ROS levels

**Figure 4:**
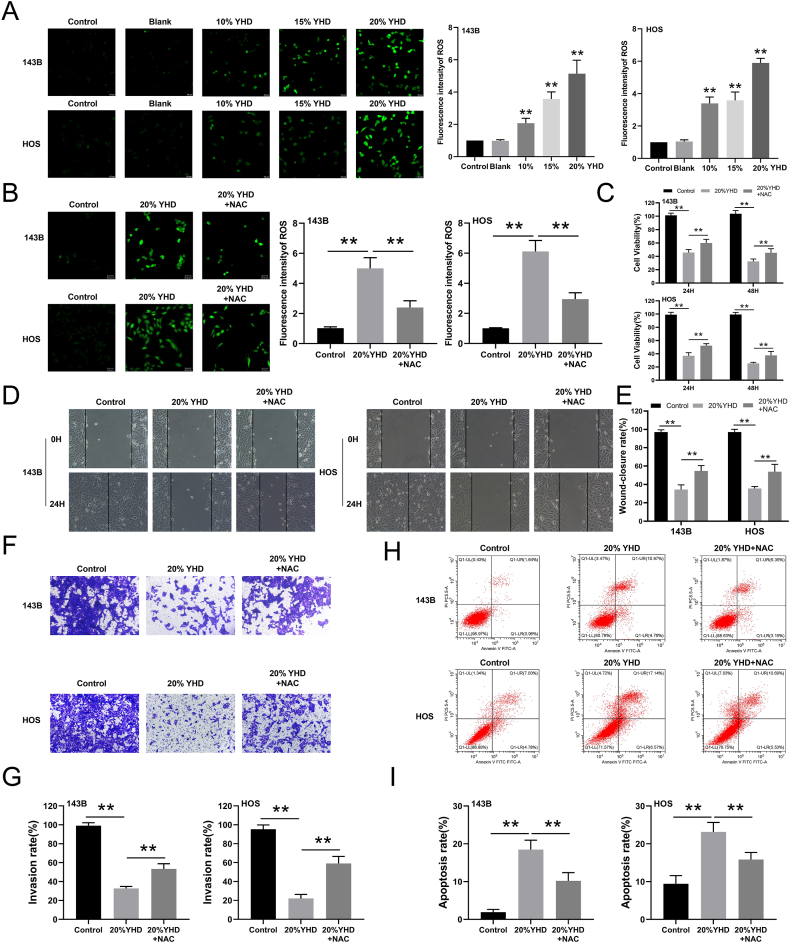
YHD induces osteosarcoma (OS) cell death by increasing ROS levels. **(A)** The effect of YHD on ROS levels in OS cells was detected using the DCFH probe method. **(B)** After N-acetylcysteine (NAC) treatment, the effect of YHD on ROS levels in OS cells was detected using the DCFH probe method. **(C)** After NAC treatment, CCK8 assay detected the effect of YHD on the viability of OS cells at 24 h and 48 h. **(D, E)** After NAC treatment, scratch healing assay showed that YHD inhibited the migration of OS cells. **(F, G)** After NAC treatment, Transwell assay showed that YHD inhibited the invasion of OS cells. **(H, I)** After NAC treatment, flow cytometry was used to detect the effect of YHD on the apoptosis of OS cells. Data were presented as mean ± standard deviation (*n* = 3). ∗*p* < 0.05 and ∗∗*p* < 0.01 versus the blank group.

In our previous studies, KEGG and GO analyses indicate that the effects of YHD on OS cells are enriched in the ROS signaling pathway. ROS, including superoxide anion (O_2_^-^), hydrogen peroxide (H_2_O_2_), and hydroxyl radical (OH-), can promote DNA damage and cell death when produced in excess, thereby affecting cellular biological behavior.^18^ We conducted fluorescence staining experiments to detect the generation of ROS within cells. The results showed that after 48 h of YHD treatment, the ROS levels in OS cells significantly increased (Fig. 4A). To verify the critical role of ROS in the anti-tumor effect of YHD, we used the antioxidant N-acetylcysteine (NAC) to inhibit ROS production. After adding NAC, we observed that the increase in ROS induced by YHD treatment was significantly reversed (Fig. 4B). Through CCK8 assays, we found that NAC partially reversed the inhibitory effect of YHD on OS cell proliferation (Fig. 4C). Additionally, through scratch healing and Transwell assays, we found that NAC partially reversed the inhibitory effects of YHD on OS cell migration and invasion (Fig. 4D–G). Finally, flow cytometry revealed that NAC partially reversed the pro-apoptotic effects of YHD on OS cells (Fig. 4H and I). Overall, these results suggest that YHD may exert its anti-OS effects by increasing intracellular ROS levels, affecting cell proliferation, migration, and apoptosis.

### YHD induces mitochondrial dysfunction in OS cells

**Figure 5:**
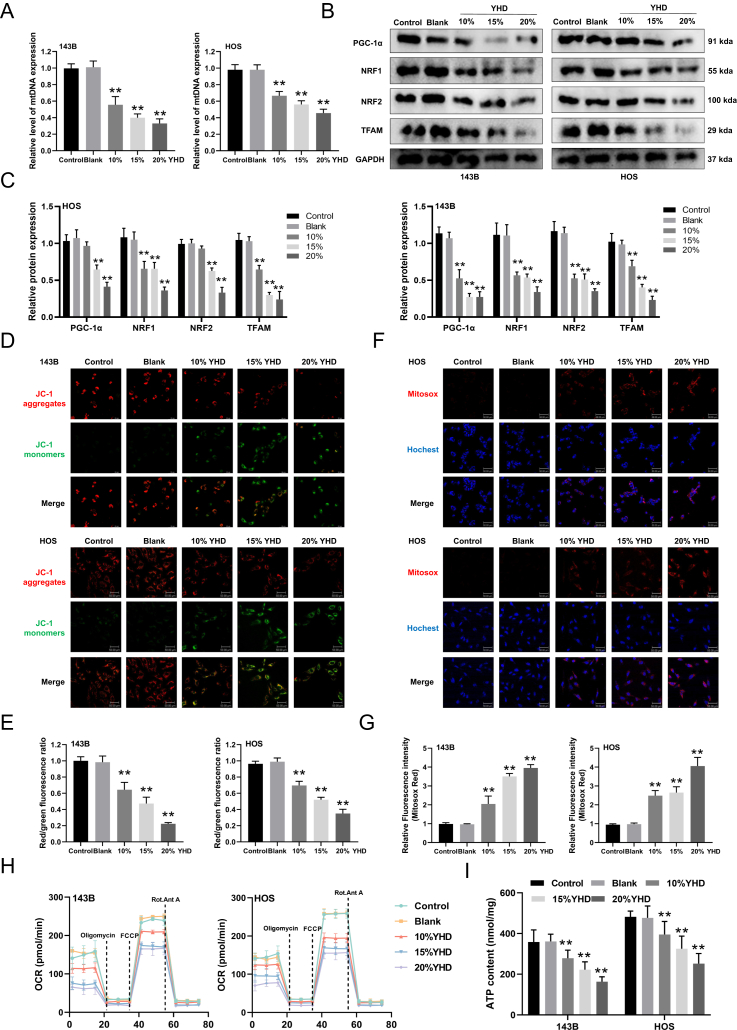
YHD can induce mitochondrial dysfunction in osteosarcoma (OS) cells. **(A)** Real-time quantitative PCR was used to measure mitochondrial DNA (mtDNA) levels. **(B, C)** Western blot analysis detected the effect of YHD on the expression levels of proteins related to mitochondrial biogenesis in OS cells. **(D, E)** JC-1 staining detected the effect of YHD on the mitochondrial membrane potential in OS cells. **(F, G)** MitoSOX staining detected the effect of YHD on mitochondrial ROS levels in OS cells. **(H)** Seahorse XFe24 analyzer measured the effect of YHD on the oxygen consumption rate (OCR) in OS cells. **(I)** The effect of YHD on ATP content in OS cells. Data were presented as mean ± standard deviation (*n* = 3). ∗*p* < 0.05 and ∗∗*p* < 0.01 versus the blank group.

Our previous results indicate that YHD can trigger the mitochondrial apoptosis pathway. Next, we explored the effects of YHD on mitochondrial function in OS cells. We first measured changes in mitochondrial DNA copy numbers after YHD treatment using real-time quantitative PCR and found a significant decrease in mitochondrial DNA abundance (Fig. 5A). We then examined the expression of mitochondrial biogenesis-related proteins (PGC-1α, NRF1, NRF2, and TFAM) and found that YHD treatment led to a reduction in the expression of these proteins in OS cells (Fig. 5B and C). Subsequently, we used JC-1 staining to observe the effects of YHD on mitochondrial membrane potential in OS cells. Normally, JC-1 exists as aggregates in functional mitochondria emitting red light, but in dysfunctional mitochondria, JC-1 exists as monomers emitting green light.^19^ Our results indicated that YHD significantly disrupted the stability of the mitochondrial membrane potential in OS cells (Fig. 5D and E). We previously confirmed that YHD could induce ROS production in OS cells. To verify whether these ROS originated from mitochondria, we conducted a MitoSOX staining experiment. The results showed that mitochondrial ROS production increased with the concentration of YHD in OS cells (Fig. 5F and G). Mitochondria play a crucial role in cellular energy supply and are involved in energy metabolism. To explore the impact of YHD on mitochondrial respiratory function in OS cells, we measured the OCR levels using a Seahorse XFe24 analyzer and found that YHD inhibited basal respiration, ATP-linked respiration, maximal respiration, and spare respiratory capacity (Fig. 5H). We also measured ATP production and obtained similar results (Fig. 5I). Overall, these results suggest that YHD induces mitochondrial dysfunction in OS cells by reducing mitochondrial DNA abundance, disrupting mitochondrial membrane potential, inhibiting ATP production, and increasing intracellular ROS production, thereby promoting cell death.

### YHD exerts anti-tumor effects on OS cells through the PI3K/AKT and p38 signaling pathways

**Figure 6:**
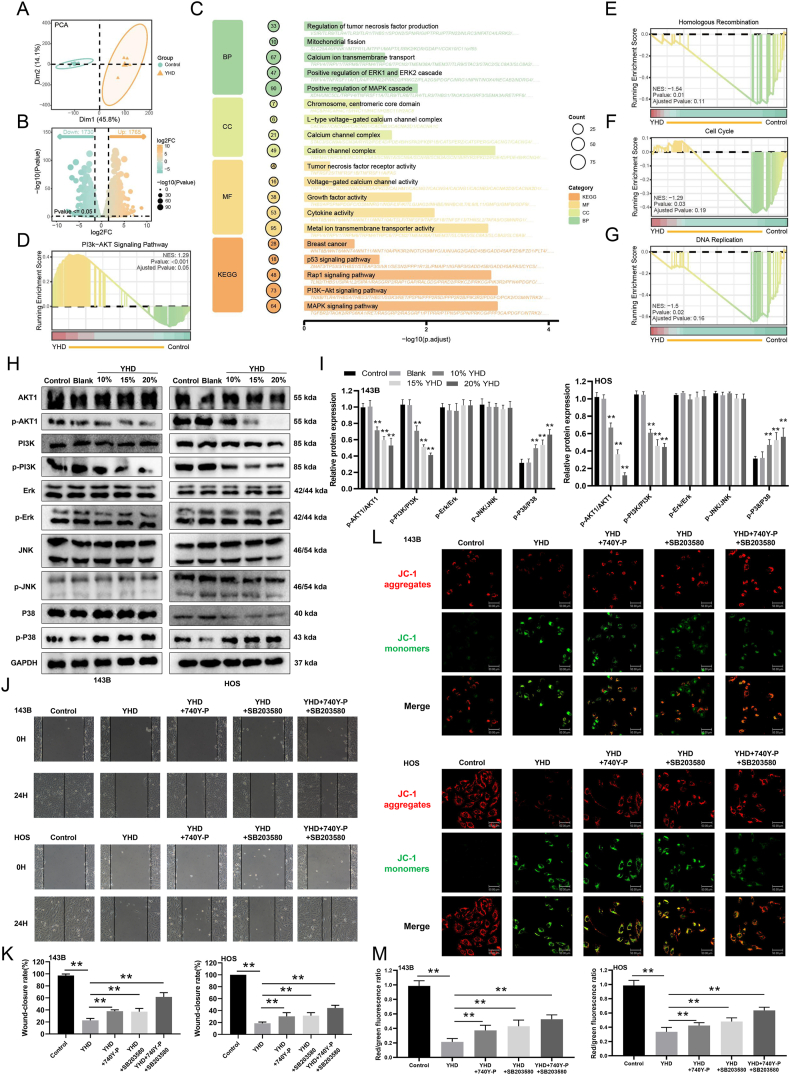
YHD exerts anti-tumor effects on osteosarcoma (OS) cells through the PI3K/AKT and p38 signaling pathways. **(A)** Principal component analysis revealed a clear distinction in gene expression profiles between the control and YHD groups. **(B)** Volcano plot identified 3495 differentially expressed genes in the YHD group. **(C)** Gene Ontology (GO) and Kyoto Encyclopedia of Genes and Genomes (KEGG) enrichment analyses. **(D**–**G)** Gene Set Enrichment Analysis (GSEA) of control and YHD groups. **(H, I)** Western blot analysis detected the effect of YHD on proteins related to the PI3K/AKT and MAPK pathways in OS cells. **(J, K)** After the addition of a PI3K activator and a P38 inhibitor, scratch healing assay showed that YHD inhibited the migration of OS cells. **(L, M)** After the addition of a PI3K activator and a P38 inhibitor, JC-1 staining detected the effect of YHD on the mitochondrial membrane potential in OS cells. Data were presented as mean ± standard deviation (*n* = 3). ∗*p* < 0.05 and ∗∗*p* < 0.01 versus the blank group.

RNA sequencing analysis was used to identify differentially expressed genes and signaling pathways between the control and YHD groups. Principal component analysis results indicated a significant difference between the two groups, suggesting that YHD altered the gene expression profile of OS cells (Fig. 6A). Differential expression analysis revealed a total of 3495 differentially expressed genes in the YHD group compared with the control group, including 1765 up-regulated and 1730 down-regulated genes (Fig. 6B). GO and KEGG enrichment analyses showed that these genes were mainly enriched in the PI3K–AKT pathway, MAPK pathway, and cation channel-related pathways (Fig. 6C). Finally, GSEA demonstrated that the PI3K–AKT signaling pathway was significantly up-regulated in the YHD group, while pathways related to homologous recombination, cell cycle, and DNA replication were significantly down-regulated (Fig. 6D–G). Next, we conducted Western blot analysis to determine if YHD treatment indeed affected the expression of the PI3K/AKT and MAPK signaling pathways in OS cells. We found that YHD significantly inhibited the expression of p-PI3K and p-AKT, but it had no effect on the total expression of PI3K and AKT (Fig. 6H and I). Further exploring the effects on the MAPK pathway, we found that YHD did not affect the expression of p-ERK or p-JNK but up-regulated the expression of p-p38, suggesting that the p38 pathway may be activated by YHD (Fig. 6H and I). To further investigate the relevance of the PI3K/AKT and P38 signaling pathways in YHD inhibition of OS, we conducted experiments using the PI3K activator 740Y–P and the P38 inhibitor SB203580. The experimental results indicated that the inhibitory effect of YHD on the proliferation of OS cells was partially reversed (Fig. S3A and S3B). Additionally, we found that the inhibitory effect of YHD on the migration (Fig. 6J and K) and invasion (Fig. S3C–S3E) of OS cells was also partially reversed. At the same time, we also observed that the ROS production in OS cells (Fig. S3F–S3H) and mitochondria (Fig. 8L and M) induced by YHD was inhibited to some extent. Overall, these data further confirm that YHD exerts its anti-OS effects by modulating the PI3K/AKT and p38 signaling pathways.

**Figure 7:**
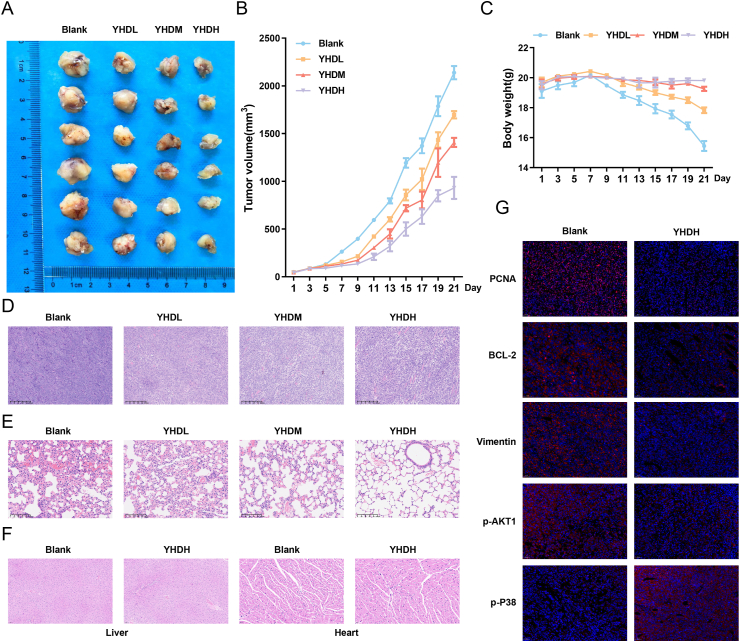
YHD inhibits the growth of OS *in vivo*. **(A)** The effect of YHD on tumorigenesis in nude mice. **(B)** The effect of YHD on tumor volume. **(C)** The effect of YHD on mouse weight. **(D)** Hematoxylin-eosin staining of the mouse tumor tissue. **(E)** Hematoxylin-eosin staining of the mouse lung tissue. **(F)** PCNA, Bcl-2, Vimentin, p-AKT, and p-P38 of the mouse tumor tissue were detected by immunohistochemistry. **(G)** Hematoxylin-eosin staining of the mouse heart and liver tissue. Data were presented as mean ± standard deviation (*n* = 6).

**Figure 8:**
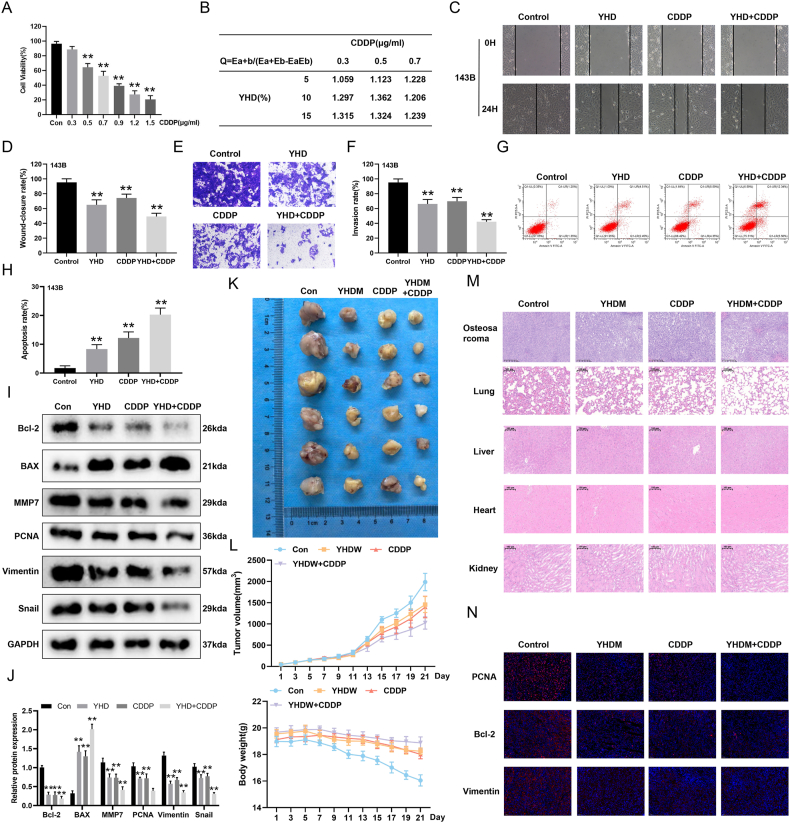
YHD combined with cisplatin (CDDP) has a synergistic inhibitory effect on the growth of OS cells. **(A)** CCK8 assay detected the effect of CDDP on the viability of OS cells at 24 h. **(B)** Combined index Q of YHD and DDP. **(C, D)** Scratch healing assay detected the effect of the combination of CDDP and YHD on OS cell migration. **(E, F)** Transwell assay detected the effect of the combination of CDDP and YHD on OS cell invasion. **(G, H)** Flow cytometry was used to detect the effect of the combination of CDDP and YHD on apoptosis in OS cells. **(I, J)** Western blot analysis detected the effect of the combination of CDDP and YHD on the expression levels of related proteins in OS cells. **(K)** The effect of the combination of CDDP and YHD on tumorigenesis in nude mice. **(L)** The effect of the combination of CDDP and YHD on tumor volume and body weight in nude mice. **(M)** Hematoxylin-eosin staining detected the effect of the combination of CDDP and YHD on tumor tissue, lung tissue, liver, heart, and kidneys in nude mice. **(N)** PCNA, Bcl-2, and Vimentin of the mouse tumor tissue were detected by immunohistochemistry. Data were presented as mean ± standard deviation (*n* = 3 for A–J; *n* = 6 for K–N). ∗*p* < 0.05 and ∗∗*p* < 0.01 versus the control group.

### YHD inhibits the growth of OS *in vivo*

To further explore the potential anti-OS effects of YHD, we established an orthotopic OS model using OS cells for experimental research. We found that the tumor volume in the YHD treatment group was significantly smaller than that in the untreated control group, demonstrating that YHD has a pronounced inhibitory effect on tumor growth (Fig. 7A and B). Monitoring the body weight of the mice showed no significant changes in the YHD treatment group (Fig. 7C), indicating that YHD might have low toxicity and minimal interference with normal physiological functions. We further analyzed the cellular morphology of the tumor tissues using hematoxylin-eosin staining. In the control group, tumor cells exhibited typical malignant features, such as larger nuclei with deeper staining and a higher nucleus-to-cytoplasm ratio. In contrast, the YHD-treated group showed a significant reduction in the nucleus-to-cytoplasm ratio and an increase in nuclear condensation and fragmentation (Fig. 7D). Hematoxylin-eosin staining of lung tissues also indicated that YHD reduced the metastatic foci in the lungs (Fig. 7E). This further confirms that YHD not only inhibits the growth of the primary tumor but also effectively reduces distant metastasis. Furthermore, hematoxylin-eosin staining of the heart and liver tissues of the mice showed no significant changes after YHD treatment (Fig. 7F), providing further evidence for the safety of YHD. Immunohistochemical staining revealed that YHD significantly reduced the expression of several protein markers associated with tumor proliferation and metastasis, such as PCNA, Bcl-2, Vimentin, and p-AKT1, while enhancing the activity of the inhibitory signal molecule p-p38. This suggests that YHD may inhibit the proliferation, invasion, and migration of OS by regulating the expression of these key signaling proteins, thereby effectively inhibiting the growth of OS cells *in vivo* (Fig. 7G). In summary, these research results indicate that YHD shows strong potential as an anti-tumor treatment option.

### YHD combined with CDDP exhibits synergistic effects on OS cells

CDDP is widely used as a first-line chemotherapy drug for OS in clinical practice. First, we used the CCK-8 assay to detect the inhibitory effect of CDDP on 143B cells. We found that CDDP also inhibited the viability of OS cells in a dose-dependent manner, with the IC50 value for 24 h calculated to be 0.7076 μg/mL (Fig. 8A). Based on the CCK-8 results, the IC50 value for YHD treatment on OS cells at 24 h was calculated to be 16.19%. To determine whether the combined use of CDDP and YHD has a synergistic effect on OS cells, we performed combination experiments using different concentrations of CDDP and YHD. We calculated the *Q* value for the combined use using Jin's formula, where E_a_ represents the inhibition rate of YHD alone, E_b_ represents the inhibition rate of CDDP alone, and E_a + b_ represents the inhibition rate of the combination of the two drugs (Fig. 8B). By calculating the *Q* value, we found that the combination of YHD and CDDP had a significant synergistic effect on OS cells (*Q* > 1.15). These results indicate that we can achieve anti-tumor effects with lower concentrations of the drugs. Therefore, we selected 10% YHD and 0.5 μg/mL CDDP for subsequent experiments. We used scratch healing assay and Transwell assay to evaluate the effects of the combination of YHD and CDDP on the migration and invasion capabilities of 143B cells. The results showed that the combined treatment significantly inhibited the migration and invasion abilities of 143B cells, with the effect being significantly better than that of single-drug treatments (Fig. 8C–F). Furthermore, we analyzed cell apoptosis using flow cytometry. The results indicated that the combined treatment more effectively promoted apoptosis in 143B cells compared with YHD or CDDP alone (Fig. 8G and H). Western blotting results revealed that the combined treatment significantly altered the expression of protein markers related to proliferation, migration, invasion, and apoptosis compared with single-drug treatments (Fig. 8I and J). Additionally, in an orthotopic OS model, we observed that the combined treatment more effectively inhibited tumor growth compared with YHD or CDDP alone, while maintaining stable body weight in the mice (Fig. 8K and L). Hematoxylin-eosin staining results showed that the combination treatment significantly reduced the malignant phenotype of tumor tissues, decreased the number and size of lung metastatic foci, and did not cause significant abnormalities in the morphology of major organs, such as the liver, kidneys, and heart. This indicates that mice tolerated the combination of YHD and CDDP well without significant toxic reactions (Fig. 8M). Finally, immunohistochemical staining results indicated that the combined treatment significantly reduced the expression of genes related to proliferation, migration, and apoptosis in tumor tissues (Fig. 8N). In summary, the combination of YHD and CDDP exhibits synergistic effects in inhibiting the growth and metastasis of OS and shows good safety, providing new insights and experimental evidence for the combined treatment of OS.

## Discussion

OS is characterized by early metastasis, poor prognosis, and a low five-year survival rate.^1^ YHD is composed of seven Chinese medicinals, which have the functions of “warming Yang” and “dispelling cold” based on the fundamental theories of traditional Chinese medicine, anti-inflammatory effect, and anti-tumor effect.^13^ This study is based on network pharmacology methods to predict the active ingredients and potential mechanisms of YHD in treating OS. Further validation was conducted in cell and mouse models to evaluate the effects and mechanisms of YHD on OS. Our study demonstrates that YHD induces ROS increase and mitochondrial dysfunction mediated by the PI3K/AKT and P38 pathways, and inhibits the proliferation, migration, and invasion of OS cells.

Using ultra-performance liquid chromatography–tandem mass spectrometry, we identified 67 major compounds and screened out 101 intersecting genes related to OS. Further GO and KEGG analyses revealed that these genes were primarily enriched in the PI3K/AKT and MAPK pathways. Based on these results, we constructed a “component-target-pathway-disease” network diagram and identified the main active components in YHD, such as (−)-epicatechin and aucubin. Molecular docking analysis showed that these active components had good binding affinity with the core targets of the PI3K/AKT and MAPK pathways, indicating potential therapeutic effects. Subsequently, we experimentally validated the potential targets and core pathway proteins identified through network pharmacology, providing strong evidence for the preliminary exploration of YHD in the treatment of OS.

The ability of tumor cells to proliferate indefinitely under favorable conditions is considered a pathogenic factor in cancer.^20^^,^^21^ Our study demonstrates that YHD can inhibit the viability and colony formation ability of OS cells, while also suppressing the expression of the proliferation-related protein PCNA. It is noteworthy that YHD does not affect the normal growth of human normal cells when treated with the same concentration, indicating that YHD has lower toxicity. Additionally, cell proliferation is achieved through the cell cycle, where each G0/G1, S, and G2/M checkpoint is strictly controlled.^22^ Inducing cell cycle arrest in tumor cells is also one of the important mechanisms by which chemotherapy drugs exert anti-tumor effects.^23^ In this study, YHD significantly increases the proportion of G2 phase in OS cells and decreases the expression of the G2/M phase arrest-related protein cyclin B, indicating cell cycle arrest at the G2 phase, inability to enter the M phase, and consequently inhibition of proliferation. Overall, YHD can inhibit the proliferation of OS cells and block their cell cycle.

OS is highly invasive and prone to distant metastasis, which is an important reason for the poor prognosis of patients. MMPs can play a direct role in the metastasis of OS.^24^ It can degrade matrix proteins, open channels, let OS cells pass through the basement membrane and enter the vascular system, and promote the invasion and migration of tumor cells.^25^^,^^26^ In addition, EMT can lead to the loss of cell polarity, the destruction of cell–cell connections, the degradation of the basement membrane, and the reorganization of the extracellular matrix, and increase the ability of tumor migration and invasion.^27^^,^^28^ In our experiments, we found that YHD could inhibit the expression of MMP2, MMP7, and MMP9 in OS cells, while also suppressing the expression of EMT-related proteins Snail, Vimentin, and N-cadherin. In *in vivo* experiments, we found that mice in the blank group had significant pulmonary metastases, while high concentrations of YHD significantly reduced the size of pulmonary metastases. Therefore, YHD may inhibit the migration and invasion of OS cells by modulating MMPs and EMT.

Apoptosis is a process of self-destruction controlled by cells autonomously.^29^ However, as the “powerhouse” of the cell, the role of mitochondria in inducing cell apoptosis cannot be ignored.^30^ During apoptosis, interruption of the mitochondrial respiratory chain electron transport leads to the generation of free radicals, lack of energy supply, blocked cell metabolism, and ultimately clearance by the organism.^31^^,^^32^ The mitochondrial pathway is mainly controlled by Bcl-2 family proteins.^33^ Through the interaction of pro-apoptotic protein Bax and anti-apoptotic protein Bcl-2, it affects the permeability of mitochondrial membrane, and then triggers the release of apoptosis related substance Cyt-C, which activates the upstream apoptosis initiator caspase-9, causes the downstream effector caspase-3 to be activated, and finally induces apoptosis by cleaving the corresponding substrate (poly (ADP-ribose) polymerase, PAPR).^34^, ^35^, ^36^ In our study, we found that YHD increased the expression of Cyt-c in OS cells, activated caspase-9, caspase-3, and Parp, while reducing the expression of Bcl-2. We speculate that YHD may induce apoptosis of OS cells by activating the intrinsic mitochondrial pathway.

Mitochondria are the primary source of ROS and the main site controlling apoptosis.^32^ Under the influence of signaling stimuli, ROS induce the opening of mitochondrial permeability transition pore, influx of Ca^2+^, and uncoupling of the mitochondrial electron transport chain, further reducing the membrane potential and prompting mitochondria to release more ROS.^37^^,^^38^ The accumulation of ROS can induce the death of tumor cells.^39^ We found that YHD could increase the level of ROS in OS cells. Importantly, when we used the antioxidant NAC to inhibit ROS production in cells, the inhibitory effect of YHD on OS growth was partially counteracted. Additionally, we explored whether YHD would cause mitochondrial dysfunction in OS cells. Experimental results showed that YHD could reduce mitochondrial abundance, disrupt mitochondrial membrane potential, and hinder ATP production in OS cells, thereby exerting its anti-OS effects.

To further clarify the molecular mechanism of YHD in treating OS, we first used network pharmacology analysis to screen potential targeting pathways, and found that the enrichment degree of PI3K/AKT and MAPK pathways was high. The PI3K/AKT signaling pathway plays a crucial role in the physiological and pathological processes of cells. Increasing evidence suggests that the factors of the PI3K/AKT signaling pathway are aberrantly expressed in human tumors, including OS.^40^^,^^41^ Immunohistochemical analysis of primary OS cases reveals a close correlation between activation of the PI3K/AKT signaling pathway and poor prognosis of the tumor.^42^ P38 MAPK is a serine/threonine kinase involved in regulating various cellular biological behaviors of tumor cells. In particular, the p38 MAPK pathway can promote cancer cell invasion by up-regulating the activity of MMP-2 and MMP-9.^43^ In addition, studies have shown that ROS can sustainably activate p38 MAPK by activating MAPK kinases and inhibiting MAPK phosphatases.^44^ Our research findings indicate that YHD can down-regulate the phosphorylation levels of PI3K and AKT and up-regulate the phosphorylation level of P38, but has no significant effect on the levels of ERK and JNK. Interestingly, the biological activity of OS cells was reversed after using a PI3K activator and a p38 inhibitor. We speculate that YHD may inhibit the growth of OS through the PI3K/Akt and p38 signaling pathways.

CDDP usually shows high sensitivity when applied to OS cells.^45^ However, due to its dose-dependent toxicity, low efficiency, and poor specificity in the treatment process, cancer cells gradually develop resistance, and the therapeutic effect is inhibited.^46^^,^^47^ Therefore, we investigated the role of YHD combined with CDDP in the treatment of OS. Research results indicate that the combination of CDDP and YHD can inhibit the proliferation, migration, and invasion of OS cells while promoting their apoptosis. Additionally, it has a significant inhibitory effect on the growth of orthotopic OS in nude mice. It can be seen that YHD enhances the sensitivity of OS cells to CDDP, achieving a synergistic effect with reduced toxicity. Given that YHD is unlikely to be used as a monotherapy in clinical practice, its role as an adjunct to enhance the sensitivity of OS cells to standard chemotherapeutic agents, such as cisplatin, warrants further investigation in future studies.

## Conclusion

Our research indicates that YHD can significantly suppress the biological processes of OS cells, and its regulatory effect may be associated with the PI3K/AKT and P38 pathways. It is noteworthy that YHD induces apoptosis of OS cells by increasing ROS levels, while also inducing mitochondrial dysfunction in OS cells. Additionally, our results indicate that YHD enhances the activity of CDDP in inhibiting OS cells, significantly suppressing their invasive capacity, exhibiting a synergistic effect between the two drugs. In summary, our research provides experimental evidence and a theoretical basis for identifying targets and drugs for treating OS, offering scientific support for the integration of traditional Chinese medicine and modern medicine in the treatment of OS.

## CRediT authorship contribution statement

**Yanran Huang:** Writing – original draft, Methodology, Investigation, Formal analysis, Conceptualization. **Dagang Tang:** Validation, Methodology, Investigation, Formal analysis. **Runhan Zhao:** Software, Data curation, Formal analysis. **Jun Zhang:** Formal analysis. **Xiao Qu:** Investigation. **Ningdao Li:** Writing – review & editing, Formal analysis. **Yi Ren:** Writing – review & editing, Supervision, Project administration. **Xiaoji Luo:** Writing – review & editing, Supervision, Project administration, Funding acquisition.

## Ethics declaration

All animal experiments employed in our study were approved by the Animal Ethics Committee of Chongqing Medical University (No. IACUC-CQMU-2024-04063).

## Data availability

The datasets used and/or analyzed during the current study are available from the corresponding author on reasonable request.

## Funding

This research was supported by the First Affiliated Hospital of Chongqing Medical University's "Discipline Summit Plan” (Chongqing, China) (No. cyyy-xkdfjhcgzh-202303), Chongqing Young and Middle-aged Medical High-end Talent Studio (China) (No. cstc2022ycjh-bgzxm0103), the Science and Technology Research Program of Chongqing Municipal Education Commission (China) (No. KJZD-M202402802), and the Horizontal Scientific Research Project of the First Affiliated Hospital of Chongqing Medical and Pharmaceutical College (Chongqing Medical and Pharmaceutical College Of China) (No. 2025YGZKY01).

## Conflict of interests

The authors declared no conflict of interests.

## Figures and Tables

**figs1:**
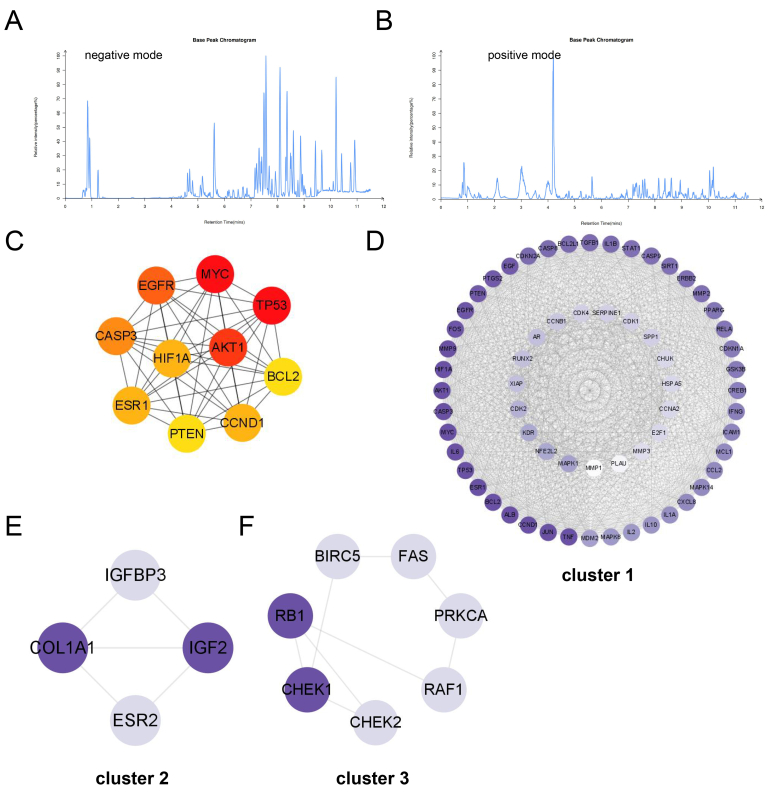


**figs2:**
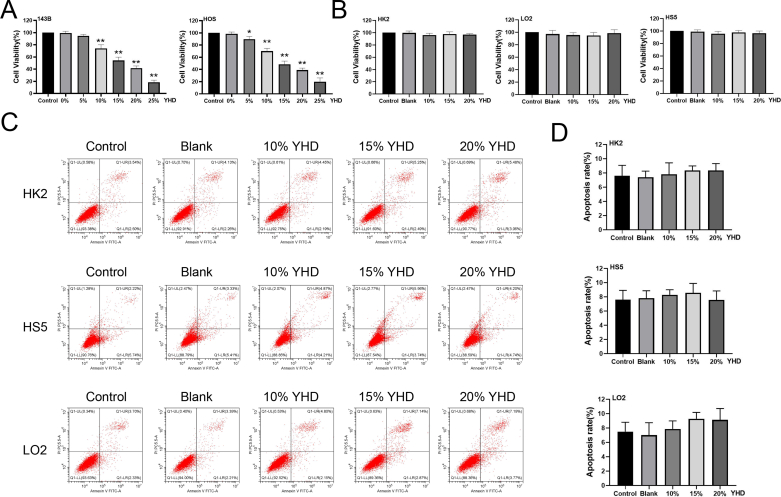


**figs3:**